# Deep Learning-Based Quantification of Visceral Fat Volumes Predicts Posttransplant Diabetes Mellitus in Kidney Transplant Recipients

**DOI:** 10.3389/fmed.2021.632097

**Published:** 2021-05-25

**Authors:** Ji Eun Kim, Sang Joon Park, Yong Chul Kim, Sang-Il Min, Jongwon Ha, Yon Su Kim, Soon Ho Yoon, Seung Seok Han

**Affiliations:** ^1^Department of Internal Medicine, Korea University Guro Hospital, Seoul, South Korea; ^2^Department of Radiology, Seoul National University College of Medicine, Seoul, South Korea; ^3^Department of Internal Medicine, Seoul National University College of Medicine, Seoul, South Korea; ^4^Department of Surgery, Seoul National University College of Medicine, Seoul, South Korea; ^5^Department of Radiology, UMass Memorial Medical Center, Worcester, MA, United States

**Keywords:** artificial intelligence, body mass index, fat, deep learning, kidney transplantation, post-transplant diabetes mellitus

## Abstract

**Background:** Because obesity is associated with the risk of posttransplant diabetes mellitus (PTDM), the precise estimation of visceral fat mass before transplantation may be helpful. Herein, we addressed whether a deep-learning based volumetric fat quantification on pretransplant computed tomographic images predicted the risk of PTDM more precisely than body mass index (BMI).

**Methods:** We retrospectively included a total of 718 nondiabetic kidney recipients who underwent pretransplant abdominal computed tomography. The 2D (waist) and 3D (waist or abdominal) volumes of visceral, subcutaneous, and total fat masses were automatically quantified using the deep neural network. The predictability of the PTDM risk was estimated using a multivariate Cox model and compared among the fat parameters using the areas under the receiver operating characteristic curves (AUROCs).

**Results:** PTDM occurred in 179 patients (24.9%) during the median follow-up period of 5 years (interquartile range, 2.5–8.6 years). All the fat parameters predicted the risk of PTDM, but the visceral and total fat volumes from 2D and 3D evaluations had higher AUROC values than BMI did, and the best predictor of PTDM was the 3D abdominal visceral fat volumes [AUROC, 0.688 (0.636–0.741)]. The addition of the 3D abdominal VF volume to the model with clinical risk factors increased the predictability of PTDM, but BMI did not.

**Conclusions:** A deep-learning based quantification of visceral fat volumes on computed tomographic images better predicts the risk of PTDM after kidney transplantation than BMI.

## Introduction

Posttransplant diabetes mellitus (PTDM), a metabolic complication after kidney transplantation, occurs in 10–40% of kidney recipients depending on the patient characteristics (1–4). Because PTDM correlates with adverse outcomes such as cardiovascular events and death, it is crucial to predict PTDM precisely and manage its occurrence in advance (4–6). Several risk factors for PTDM have been identified, such as obesity (7, 8), high blood pressure (9), immunosuppressive agents (10–12), infection with hepatitis C virus (4, 13), hyperuricemia (13), and hypertriglyceridemia (13). High values of body mass index (BMI), one of the crude measures for body fat, predict the risk of DM (14), but this relationship has not necessarily happened in PTDM (4, 15–18).

BMI is a simple and convenient measure for adiposity but does not reflect body shape and fat distribution, which leads to inevitable limitations in the precise estimation of visceral fat (VF) volumes (19). Furthermore, the relationship with worse outcomes may depend on the race as Asians have a higher proportion of body fat mass for a given BMI than Caucasians (20). A bioelectrical impedance analysis, dual-energy X-ray absorptiometry, and cross-sectional computed tomography (CT) have been used to substitute BMI (21–25). Analyzing body components in cross-sectional CT imaging is regarded as a reference standard. However, its clinical use remains limited because the analysis requires a considerable amount of time and effort of specialists.

The introduction of a deep-learning algorithm in medicine attempts to change the paradigm of the clinical process (26, 27), particularly of diagnostic imaging (28). Deep learning algorithms have shown potential in automatic fat quantification on CT images and thus can reduce the laborious work involved in fat segmentation (29). Herein, we addressed whether deep-learning-based volumetric fat quantification on CT images after segmenting body fat distribution predicted the risk of PTDM more precisely than BMI.

## Methods

### Study Subjects

The study was approved by the institutional review board of the Seoul National University Hospital (no. H-1907-072-1047) and complied with the Declaration of Helsinki. Among 1,377 adults (aged ≥ 18 years) who consecutively underwent kidney transplantation at Seoul National University Hospital between 2003 and 2017, 983 patients who underwent abdominal CT scans within 1 year before transplantation were initially reviewed. Of these, 38 patients in whom the CT scan did not sufficiently cover the abdominal waist from the iliac crest to the lower margin of the ribs and 227 patients who had DM before transplantation were excluded. Accordingly, 718 patients were analyzed in the present study. Under the review board's approval, informed consent was waived.

### Data Collection and Definition

Baseline information such as age, sex, weight, height, type of pretransplant dialysis, donor type (living or deceased), ABO incompatibility, positivity for hepatitis B surface antigen and anti-hepatitis C virus antibody, the number of human leukocyte antigen mismatches, and the immunosuppressive regimens for induction (e.g., basiliximab and anti-thymocyte globulin) and maintenance (e.g., steroid, calcineurin inhibitor, and mycophenolic acid) were collected. A combination therapy of steroids, tacrolimus, and mycophenolic acid was primarily used for maintenance in our center. BMI was calculated as weight (kg)/height (m^2^). Laboratory findings such as total cholesterol, high-density lipoprotein cholesterol, triglyceride, and uric acid were collected in the fasting state before kidney transplantation. Low-density lipoprotein cholesterol was calculated using the following formula: total cholesterol – high-density lipoprotein cholesterol – (triglyceride/5).

The primary outcome was PTDM. PTDM was diagnosed when recipients needed antidiabetic medications because of high blood glucose levels. The secondary outcomes were delayed graft function (i.e., the requirement of dialysis within 7 days after transplantation) and biopsy-proven acute rejection such as acute T-cell-mediated and antibody-mediated rejections.

### Deep Learning-Based Measurement of 2D and 3D Fat Volumes

All abdominal CT scans were performed using multidetector CT scanners without the intravenous administration of contrast media. The mean interval between CT scanning and transplantation was 91.1 ± 54.5 days. After uploading precontrast volumetric abdominal CT images to commercially available segmentation software (MEDIP Deep Catch v1.0.0.0, MEDICALIP Co. Ltd., Seoul, Korea), a 3D U-Net automatically generated a volumetric mask of 7 compartments in <1.5 min with the recommended specifications (30): skin, bone, muscle, VF, subcutaneous fat (SF), internal organs with vessels, and central nervous system. The network was developed using 39,286 labeled whole-body CT images and provided an average segmentation accuracy for VF and SF of 92.4–98.9% and 94.1–99.7%, respectively, in internal and external validation datasets of whole-body CT scans. After the volumetric segmentation of VF and SF, the range of the whole abdominal waist was automatically extracted between the iliac crest and the margin of the lowest rib, with subsequent calculation of the 3D volumes of VF and SF in the whole abdominal or waist area and 2D volumes at the midpoint of the abdominal waist (31). An experienced body radiologist (SH Yoon) identified whether the results of segmentation and the range of the abdominal waist were appropriate. VF and SF volumes were summed to calculate total fat (TF) volumes. All of the TF, VF, and SF volumes were normalized by the height squared (m^2^) (32).

### Statistical Analysis

All statistical analyses were performed with the STATA (version 15.1; StataCorp, College Station, TX, USA) and R (version 3.5.0; R Core Team) software. Continuous variables are presented as the mean and standard deviation or median and interquartile ranges and compared by Student's *t*-test or the Wilcoxon rank-sum test, respectively. Categorical variables are presented as percentages and compared by the chi-squared test. Ordinary least-squares linear regression and fractional polynomial regression with continuous variables were used to determine a nonlinear relationship. Univariate and multivariable Cox regression models were applied to estimate the hazard ratio of the risks of outcomes. The Stata function *mkspline* was used to create a restricted cubic spline function to describe the hazard ratio of outcomes according to the fat parameters. The areas under the receiver operating characteristic curves (AUROCs) for predicting the risk of PTDM were compared between fat parameters using permutation tests (33, 34). The AUROCs for cumulative predictive probability depending on the follow-up duration were drawn using the *survivalROC* package in R. For the risk of delayed graft function, a multivariate logistic regression model was applied. A *P*-value of <0.05 was considered significant.

## Results

### Baseline Characteristics

The mean age was 45.2 ± 12.6 years old, and 431 patients (60.0%) were male. A total of 81.8% of patients were treated with anti-hypertensive agents. A total of 65.5% of patients received transplants from living donors. The mean preoperative BMI was 22.5 ± 3.4 kg/m^2^. Other baseline characteristics of kidney recipients are shown in Table 1.

**Table 1:** Baseline characteristics of the study subjects.

**Variables**	**Total (*n* = 718)**
Age (years)	45.2 ± 12.6
Male sex (%)	60.0
Body mass index (kg/m^2^)	22.5 ± 3.4
Deceased donor (%)	34.5
**Type of pre-transplant dialysis (%)**	
Preemptive	14.4
Hemodialysis	66.4
Peritoneal dialysis	19.2
Pre-transplant dialysis duration, months	23 [2–79]
**Cause of kidney failure (%)**	
Hypertension	9.5
Glomerulonephritis	51.7
Polycystic kidney disease	10.3
Others	28.6
Hypertension (%)	81.8
Positivity for anti-hepatitis C virus antibody (%)	2.1
Positivity for hepatitis B surface antigen (%)	6.7
ABO incompatibility (%)	9.6
Number of HLA mismatch > 3 (%)	39.7
**Induction agent (%)**	
None	11.8
Basiliximab	85.8
Anti-thymocyte globulin	2.4
**Calcineurin inhibitor (%)**	
None	2.9
Cyclosporine	8.6
Tacrolimus	88.4
Mycophenolic acid (%)	98.6
**Laboratory findings**	
Total cholesterol (mg/dL)	159.0 ± 36.7
Triglyceride (mg/dL)	123.7 ± 80.3
HDL cholesterol (mg/dL)	50.2 ± 16.5
LDL cholesterol (mg/dL)	92.4 ± 32.5
Uric acid (mg/dL)	6.0 ± 2.0

*HLA, human leukocyte antigen; HDL, high-density lipoprotein; LDL, low-density lipoprotein*.

### Fat Volume Parameters and Their Correlation With BMI

**Figure 1:**
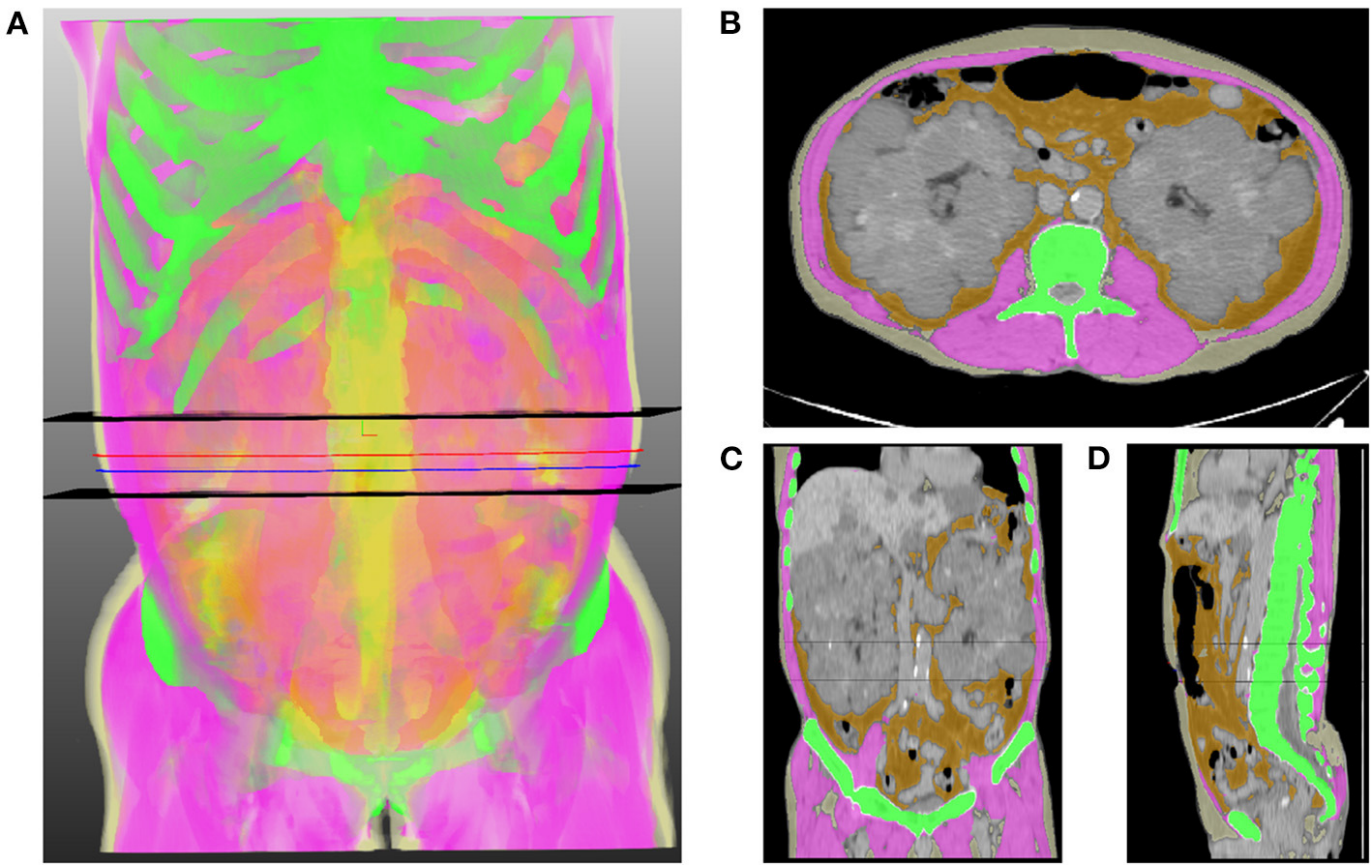
Representative image of the volumetric extraction of body compositions. **(A)** 3D translucent image shows a volumetric segmentation of muscle (pink), subcutaneous fat (light yellow), abdominal visceral fat (orange), and bone (green) using a deep neural network. Two black horizontal planes indicate the range of the abdominal waist between the lowest end of the rib cage and the uppermost end of the iliac crest. Blue and red lines indicate the levels of the umbilicus and the middle of the abdominal waist, respectively. Axial **(B)**, coronal **(C)**, and sagittal **(D)** images show the results of segmentation, which are overlaid on orthogonal cross-sectional images.

**Figure 2:**
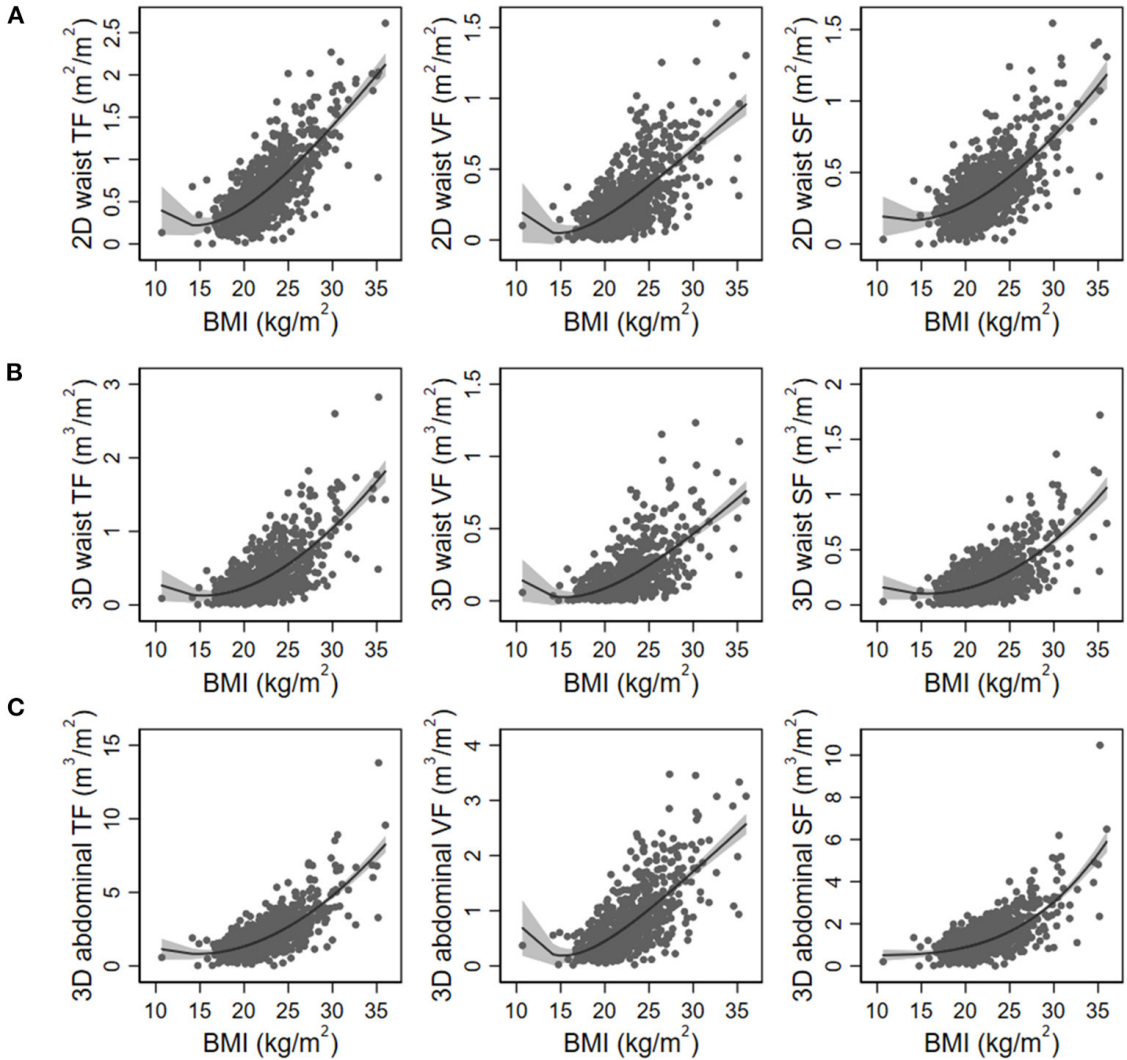
Nonlinear regression models of body mass index (BMI) with 2D waist **(A)**, 3D waist **(B)**, and 3D abdominal fat volumes **(C)**. The gray area indicates 95% confidence intervals. TF, total fat; VF, visceral fat; SF, subcutaneous fat.

Figure 1 shows the schematic diagram to measure 2D waist, 3D waist, and 3D abdominal fat volumes using the deep neural network algorithm on 3D-reconstructed CT images. The mean values of 2D waist, 3D waist, and 3D abdominal TF volumes were 0.66 ± 0.41, 0.41 ± 0.36, and 2.08 ± 1.42 m^3^/m^2^, respectively. Although all the 2D and 3D fat volumes correlated with BMI (Table 2), their coefficients of determination (*r*^2^) in linear regression models were <0.6. When the nonlinear relationship was subsequently applied, a J-shaped relationship, but not a linear one, was shown between them (Figure 2).

**Table 2:** 2D and 3D fat volumes and their correlations with body mass index.

**Parameters**	**Mean ± standard deviation**	***r*^**2**^**	***P***
2D volume of waist TF	0.66 ± 0.41 m^2^/m^2^	0.539	<0.001
2D volume of waist VF	0.28 ± 0.23 m^2^/m^2^	0.426	<0.001
2D volume of waist SF	0.38 ± 0.23 m^2^/m^2^	0.420	<0.001
3D volume of waist TF	0.41 ± 0.36 m^3^/m^2^	0.448	<0.001
3D volume of waist VF	0.17 ± 0.18 m^3^/m^2^	0.399	<0.001
3D volume of waist SF	0.24 ± 0.21 m^3^/m^2^	0.381	<0.001
3D volume of abdominal TF	2.08 ± 1.42 m^3^/m^2^	0.526	<0.001
3D volume of abdominal VF	0.76 ± 0.57 m^3^/m^2^	0.466	<0.001
3D volume of abdominal SF	1.32 ± 0.90 m^3^/m^2^	0.454	<0.001

*TF, total fat; VF, visceral fat; SF, subcutaneous fat*.

### Fat Volume Parameters and the Risk of PTDM

**Figure 3:**
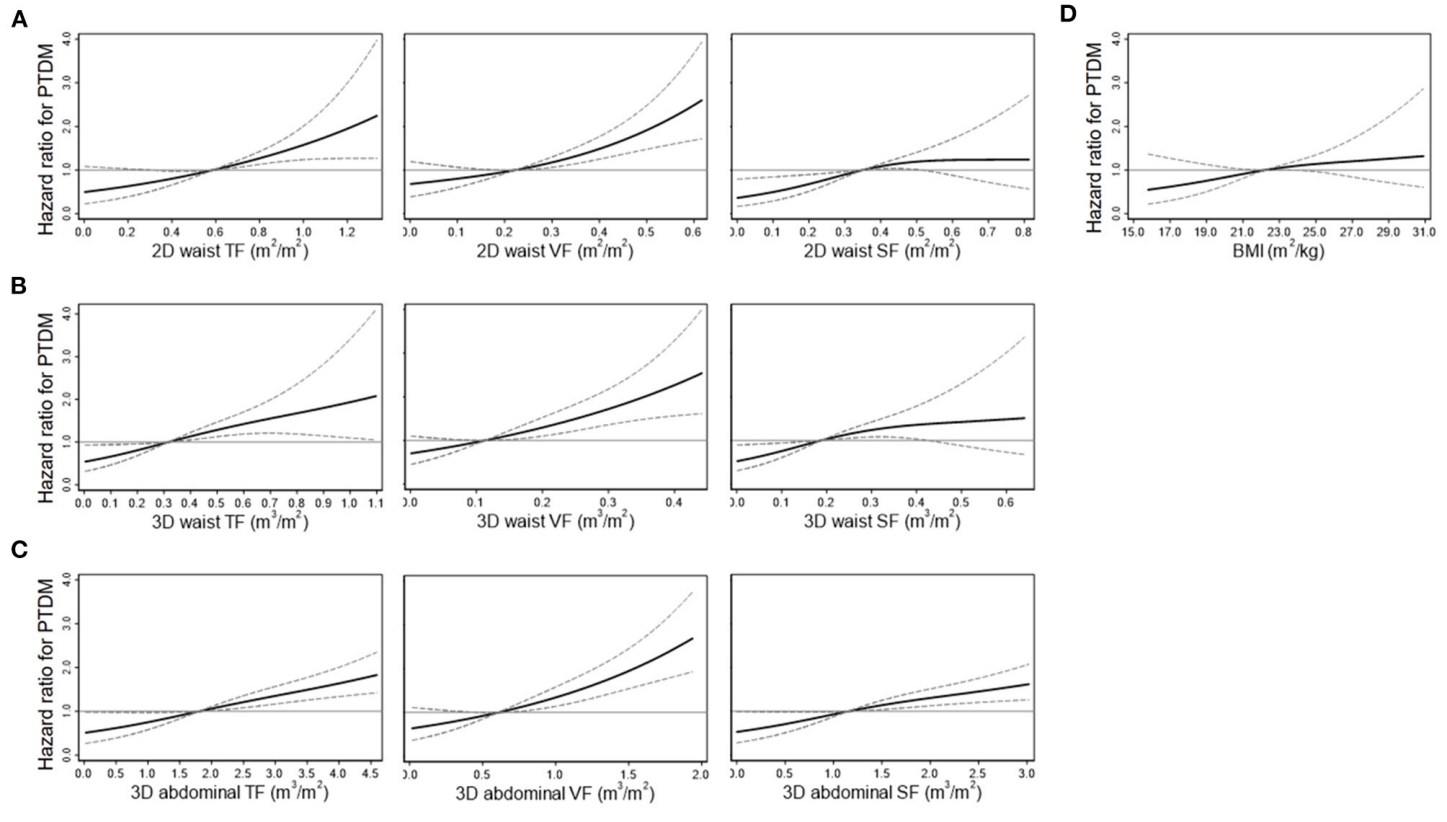
Restricted cubic spline curves for the risk of posttransplant diabetes mellitus (PTDM) according to the 2D waist **(A)**, 3D waist **(B)**, 3D abdominal fat volumes **(C)**, and body mass index (BMI) **(D)**. Curves were adjusted by multiple variables, such as age, sex, ABO incompatibility, anti-hepatitis C virus antibody, the induction agents, and serum levels of high-density lipoprotein cholesterol. Solid and dashed lines indicate hazard ratios and 95% confidence intervals, respectively. TF, total fat; VF, visceral fat; SF, subcutaneous fat.

During the median follow-up duration of 5 years (2.5–8.6 years), PTDM occurred in 179 patients (24.9%). The prevalence of PTDM was 13.2 and 18.1% at 1 year and 3 years after transplantation, respectively. Among the baseline clinical variables, age, ABO incompatibility, induction regimens, and serum levels of high-density lipoprotein cholesterol were associated with the risk of PTDM (Supplementary Table 1). All 2D and 3D fat parameters and BMI were associated with the risk of PTDM irrespective of adjustment for multiple variables (Table 3). When a nonlinear relationship was applied, the relationship with the PTDM risk seemed to be more prominent in the VF and TF volumes compared with the SF volumes and BMI (Figure 3).

**Table 3:** Risk of posttransplant diabetes mellitus according to the fat parameters.

	**Model 1**		**Model 2**		**Model 3**	
**Parameters**	**HR (95% CI)**	***P***	**HR (95% CI)**	***P***	**HR (95% CI)**	***P***
2D volume of waist TF (per 1 m^2^/m^2^)	3.71 (2.74–5.04)	<0.001	3.22 (2.31–4.48)	<0.001	3.01 (2.07–4.36)	<0.001
2D volume of waist VF (per 1 m^2^/m^2^)	8.73 (5.36–14.22)	<0.001	5.88 (3.44–10.05)	<0.001	5.74 (3.07–10.73)	<0.001
2D volume of waist SF (per 1 m^2^/m^2^)	5.64 (3.25–9.79)	<0.001	6.45 (3.50–11.90)	<0.001	4.94 (2.55–9.57)	<0.001
3D volume of waist TF (per 1 m^3^/m^2^)	3.05 (2.26–4.11)	<0.001	2.98 (2.17–4.09)	<0.001	2.47 (1.74–3.50)	<0.001
3D volume of waist VF (per 1 m^3^/m^2^)	9.45 (5.36–16.65)	<0.001	7.41 (4.04–13.62)	<0.001	6.45 (3.15–13.23)	<0.001
3D volume of waist SF (per 1 m^3^/m^2^)	5.31 (3.04–9.26)	<0.001	6.24 (3.49–11.16)	<0.001	4.02 (2.15–7.53)	<0.001
3D volume of abdominal TF (per 1 m^3^/m^2^)	1.34 (1.24–1.44)	<0.001	1.30 (1.21–1.41)	<0.001	1.24 (1.14–1.35)	<0.001
3D volume of abdominal VF (per 1 m^3^/m^2^)	2.41 (1.98–2.94)	<0.001	2.22 (1.78–2.77)	<0.001	2.10 (1.64–2.70)	<0.001
3D volume of abdominal SF (per 1 m^3^/m^2^)	1.42 (1.27–1.59)	<0.001	1.40 (1.25–1.57)	<0.001	1.29 (1.13–1.46)	<0.001
Body mass index (per 1 kg/m^2^)	1.12 (1.07–1.16)	<0.001	1.10 (1.05–1.15)	<0.001	1.08 (1.03–1.13)	0.001

*Model 1: Unadjusted*.

*Model 2: Adjusted for age and sex*.

*Model 3: Adjusted for age, sex and variables which had P <0.1 in univariate analysis (ABO incompatibility, induction agents, triglyceride level, high density lipoprotein cholesterol level and positivity for anti-hepatitis C virus antibody)*.

*HR, hazard ratio; CI, confidence interval; TF, total fat; VF, visceral fat; SF, subcutaneous fat*.

**Figure 4:**
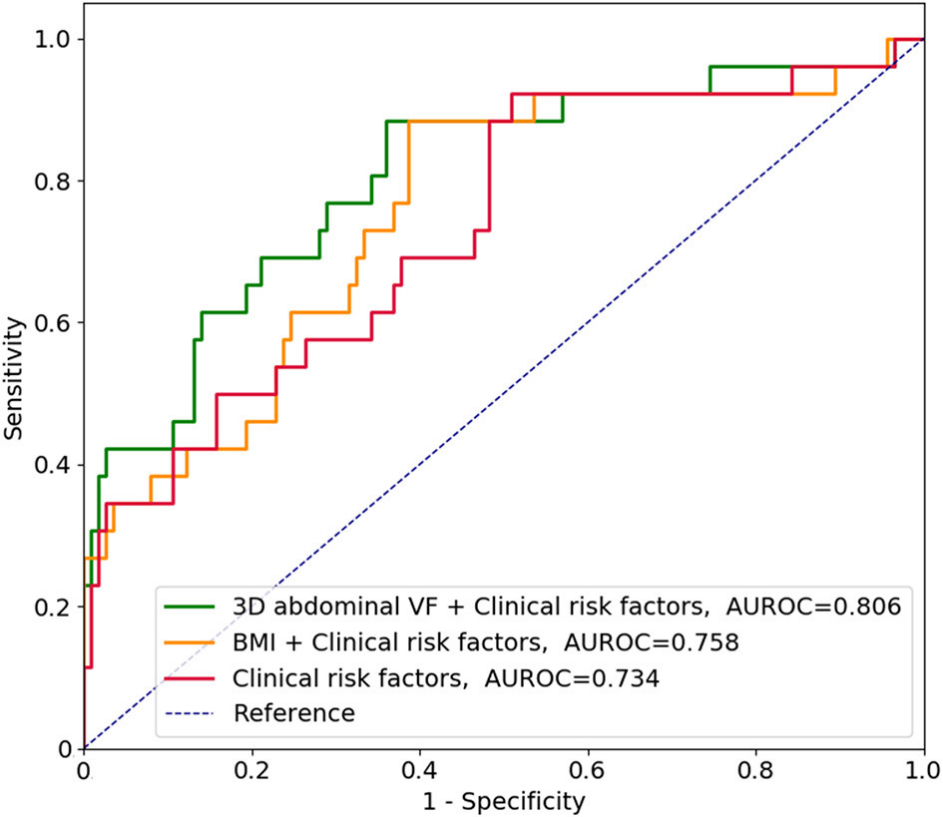
Receiver operating characteristic curves in predicting the 3-year risk of posttransplant diabetes mellitus along with clinical risk factors. According to Supplementary Table 1, clinical risk factors included age, ABO incompatibility, induction regimens, and serum levels of high-density lipoprotein cholesterol. VF, visceral fat; AUROC, area under the receiver operating characteristic curve; BMI, body mass index.

The AUROCs for predicting the 3-year risk of PTDM were higher in VF and TF volumes from 2D and 3D quantification than in BMI (Table 4). The highest value was identified in 3D abdominal VF volumes. The corresponding curves support these results (Supplementary Figure 1). When the cumulative AUROCs were evaluated, VF volumes had higher values than BMI irrespective of the follow-up period (Supplementary Figure 2). We evaluated whether the addition of fat parameters to the risk model with other clinical factors, which had *P* < 0.05 in Supplementary Table 1, increased the overall predictability for the 3-year PTDM. The 3D abdominal VF volumes elevated the predictability of the model when added (*P* = 0.015), but BMI did not (*P* = 0.206). The corresponding ROC curves support these results (Figure 4).

**Table 4:** Area under the receiver operating characteristic curves of fat parameters in predicting 3-year posttransplant diabetes mellitus.

**Parameters**	**AUROC (95% CI)**	***P***
2D volume of waist TF	0.684 (0.632–0.735)	0.001
2D volume of waist VF	0.688 (0.635–0.740)	0.001
2D volume of waist SF	0.628 (0.576–0.679)	0.532
3D volume of waist TF	0.669 (0.617–0.720)	0.023
3D volume of waist VF	0.685 (0.634–0.735)	0.002
3D volume of waist SF	0.628 (0.575–0.681)	0.561
3D volume of abdominal TF	0.672 (0.619–0.724)	0.008
3D volume of abdominal VF	0.688 (0.636–0.741)	<0.001
3D volume of abdominal SF	0.634 (0.581–0.687)	0.378
Body mass index	0.612 (0.559–0.664)	Reference

*AUROC, area under the receiver operating characteristic curve; CI, confidence interval; TF, total fat; VF, visceral fat; SF, subcutaneous fat*.

### Association With Other Transplant Outcomes

Because high fat volumes may confer the risk of rejection and delayed graft function according to previous studies (35, 36), other risks such as delayed graft function and rejections were assessed. For delayed graft function, the 3D volumes of abdominal VF and TF were predictors with odds ratios of 2.08 (1.12–3.87) and 1.33 (1.01–1.75) per 1-unit increase, respectively, but other fat parameters, including BMI, were not (Supplementary Table 2). None of the fat parameters were associated with the risk of rejections in the present cohort (Supplementary Table 3).

## Discussion

The present study used the deep learning algorithm to quantify the 2D and 3D fat volumes in pretransplant CT images and identified that their relationship with BMI was not linear. Although all the fat parameters were associated with the risk of PTDM, the predictability was greater in VF volumes than in BMI. The addition of 3D abdominal VF volume to the model with clinical risk factors increased the predictability of PTDM, but BMI did not. The present results indicate that precise quantification of fat volumes by deep learning algorithm may help to alert clinicians of the risk of PTDM.

Precise measurement of fat components is a critical issue in classifying risky patients based on obesity-related outcomes. BMI, which is based on weight and height, is a commonly used method to measure fat mass, but it does not take into account other body compositions such as muscle and bone. BMI seems to be an insufficient marker to assess PTDM based on inconsistent research results (16–18). VF components have been revealed as a risk factor for metabolic and cardiovascular diseases in the general population, independent of BMI (37). VF was related to glucose intolerance in kidney recipients (38). Based on both the previous and present results, the estimation of VF volumes is needed to predict the risk of PTDM more precisely than BMI.

Abdominal imaging methods, including CT, have been used to assess the volumes of fat components using computer calculator more than before (9, 39–41). This method has been validated in several studies, but optimization is needed to reduce bias and the time consumed by the task (42–44). The present study applied a deep learning algorithm to automatically segment the VF and SF components and exclude muscle and bone, which eventually detected the fat volumes quickly and unbiasedly for a number of images. Because kidney transplant recipients undergo abdominal CT scans for routine preoperative work-up, our approach using readily available software is implementable for more accurate prediction of PTDM than BMI, which may help in designing a plan to prevent PTDM occurrence.

Despite the valuable findings of our study, there are some limitations that need to be addressed. Waist circumference, a useful method for fat volume, was not evaluated. Follow-up CT images may be helpful to predict the risk of PTDM, but the present study could not obtain these data. Other unidentified factors, such as diet and exercise information, could have an interacting effect on the relationships observed in the study. Only Korean patients were analyzed, and no other populations were analyzed. Nevertheless, the primary purpose of the study was to address the application of the deep-learning-algorithm-based quantification of 2D and 3D fat volumes in kidney recipients, not to build a final model. A prospective application and adjustment of our algorithm to other cohorts is warranted in future studies.

Quantification of VF components with a deep learning algorithm successfully predicts PTDM, which is better than the measurement of BMI. Deep-learning-based approaches are increasingly used in many clinical aspects, and the present results will be a basis for application in the transplant field.

## Data Availability Statement

The raw data supporting the conclusions of this article will be made available by the authors, without undue reservation.

## Ethics Statement

The studies involving human participants were reviewed and approved by the Institutional Review Board of the Seoul National University Hospital. Written informed consent for participation was not required for this study in accordance with the national legislation and the institutional requirements.

## Author Contributions

JK: data analysis and interpretation and manuscript drafting. SP: technical support. YCK, S-IM, and JH: data collection. YSK: technical support and supervision. SY and SH: project development, data interpretation, supervision, and manuscript editing. All authors contributed to the article and approved the submitted version.

## Conflict of Interest

SP is the CEO of MEDICALIP Co. Ltd., Seoul, Korea. The remaining authors declare that the research was conducted in the absence of any commercial or financial relationships that could be construed as a potential conflict of interest.
