# Supplementary material for: Deep Learning-Based Quantification of Visceral Fat Volumes Predicts Posttransplant Diabetes Mellitus in Kidney Transplant Recipients
Source: Front Med (Lausanne). 2021 May 25;8:632097. doi: 10.3389/fmed.2021.632097 (PMC8185023; doi:10.3389/fmed.2021.632097)

Supplementary Material

# Supplementary Tables

**Table S1.** Cox regression model for the risk of posttransplant diabetes mellitus

| Variables | HR (95% CI) | *P* |
| --- | --- | --- |
| Age (per 1 year) | 1.05 (1.04–1.07) | < 0.001 |
| Female (vs. male) | 0.95 (0.70–1.30) | 0.751 |
| Deceased donor (vs. living) | 1.18 (0.86–1.62) | 0.315 |
| RRT type |  |  |
| Pre-emptive | Reference |  |
| Hemodialysis | 0.87 (0.56–1.34) | 0.533 |
| Peritoneal dialysis | 0.71 (0.41–1.21) | 0.204 |
| Hypertension (vs. none) | 1.09 (0.71–1.68) | 0.679 |
| Positivity for anti-hepatitis C virus antibody (vs. none) | 2.15 (0.95–4.85) | 0.066 |
| Positivity for hepatitis B surface antigen (vs. none) | 0.71 (0.36–1.39) | 0.319 |
| ABO incompatibility (vs. compatibility) | 1.80 (1.12–2.88) | 0.015 |
| Number of HLA mismatch > 3 (vs. ≤ 3) | 1.07 (0.79–1.46) | 0.660 |
| Induction agent |  |  |
| None | Reference |  |
| Basiliximab | 1.70 (1.02–2.85) | 0.043 |
| Anti-thymocyte globulin | 4.56 (1.86–11.18) | 0.001 |
| Calcineurin inhibitor |  |  |
| None | Reference |  |
| Cyclosporine | 1.64 (0.37–7.34) | 0.519 |
| Tacrolimus | 2.91 (0.72–11.74) | 0.134 |
| Mycophenolic acid (vs. none) | 0.81 (0.26–2.55) | 0.724 |
| Total cholesterol (per 1 mg/dL) | 1.00 (1.00–1.00) | 0.979 |
| Triglyceride (per 1 mg/dL) | 1.00 (1.00–1.00) | 0.063 |
| HDL cholesterol (per 1 mg/dL) | 0.99 (0.97–1.00) | 0.008 |
| LDL cholesterol (per 1 mg/dL) | 1.00 (0.99–1.00) | 0.523 |
| Uric acid (per 1 mg/dL) | 0.97 (0.90–1.04) | 0.404 |

Abbreviations: HR, hazard ratio; CI, confidence interval; RRT, renal replacement therapy; HLA, human leukocyte antigen; HDL, high-density lipoprotein; LDL, low-density lipoprotein.

**Table S2.** Risk of delayed graft function according to the fat parameters

|  | Model 1 | | Model 2 | | Model 3 | |
| --- | --- | --- | --- | --- | --- | --- |
| Parameters | OR (95% CI) | *P* | OR (95% CI) | *P* | OR (95% CI) | *P* |
| 2D volume of waist TF (per 1 m^2^/m^2^) | 2.29 (1.12–4.68) | 0.024 | 1.86 (0.87–4.02) | 0.112 | 2.26 (0.97–5.30) | 0.060 |
| 2D volume of waist VF (per 1 m^2^/m^2^) | 3.82 (1.12–13.08) | 0.033 | 2.43 (0.63–9.31) | 0.197 | 2.97 (0.70–12.70) | 0.141 |
| 2D volume of waist SF (per 1 m^2^/m^2^) | 3.33 (0.95–11.71) | 0.060 | 3.09 (0.77–12.42) | 0.112 | 4.71 (0.98–22.65) | 0.053 |
| 3D volume of waist TF (per 1 m^3^/m^2^) | 2.01 (0.94–4.29) | 0.071 | 1.76 (0.78–3.97) | 0.174 | 2.57 (0.97–6.83) | 0.058 |
| 3D volume of waist VF (per 1 m^3^/m^2^) | 4.49 (1.02–19.74) | 0.047 | 3.08 (0.63–15.19) | 0.166 | 4.59 (0.78–27.09) | 0.092 |
| 3D volume of waist SF (per 1 m^3^/m^2^) | 2.68 (0.69–10.41) | 0.156 | 2.45 (0.56–10.82) | 0.236 | 5.67 (0.89–36.10) | 0.066 |
| 3D volume of abdominal TF (per 1 m^3^/m^2^) | 1.19 (0.99–1.44) | 0.062 | 1.14 (0.93–1.40) | 0.207 | 1.33 (1.01–1.75) | 0.042 |
| 3D volume of abdominal VF (per 1 m^3^/m^2^) | 1.84 (1.15–2.97) | 0.012 | 1.62 (0.96–2.75) | 0.071 | 2.08 (1.12–3.87) | 0.020 |
| 3D volume of abdominal SF (per 1 m^3^/m^2^) | 1.20 (0.91–1.60) | 0.201 | 1.14 (0.83–1.57) | 0.403 | 1.45 (0.93–2.28) | 0.105 |
| Body mass index (per 1 kg/m^2^) | 1.05 (0.96–1.16) | 0.281 | 1.04 (0.94–1.15) | 0.487 | 1.06 (0.95–1.19) | 0.275 |

Model 1: Unadjusted.

Model 2: Adjusted for age and sex.

Model 3: Adjusted for age, sex and variables which had *P* < 0.1 in univariate analysis (deceased donor, pre-transplant dialysis duration, ABO incompatibility, HLA mismatch and induction agents).

Abbreviations: OR, odds ratio; CI, confidence interval; TF, total fat; VF, visceral fat; SF, subcutaneous fat.

**Table S3.** Risk of biopsy-proven acute rejection according to the fat parameters

|  | Acute rejection | | Acute T cell-mediated rejection | | Acute antibody-mediated rejection | |
| --- | --- | --- | --- | --- | --- | --- |
| Parameters | HR (95% CI)* | *P* | HR (95% CI)* | *P* | HR (95% CI)* | *P* |
| 2D volume of waist TF (per 1 m^2^/m^2^) | 1.12 (0.86–1.44) | 0.401 | 1.07 (0.82–1.39) | 0.618 | 1.52 (0.82–2.83) | 0.183 |
| 2D volume of waist VF (per 1 m^2^/m^2^) | 1.20 (0.76–1.87) | 0.436 | 1.09 (0.69–1.73) | 0.715 | 2.42 (0.77–7.62) | 0.131 |
| 2D volume of waist SF (per 1 m^2^/m^2^) | 1.19 (0.75–1.88) | 0.467 | 1.14 (0.71–1.83) | 0.594 | 1.67 (0.56–4.97) | 0.359 |
| 3D volume of waist TF (per 1 m^3^/m^2^) | 1.08 (0.81–1.43) | 0.597 | 0.96 (0.71–1.29) | 0.774 | 1.56 (0.86–2.83) | 0.140 |
| 3D volume of waist VF (per 1 m^3^/m^2^) | 1.14 (0.66–1.98) | 0.639 | 0.90 (0.50–1.62) | 0.727 | 2.51 (0.69–9.09) | 0.162 |
| 3D volume of waist SF (per 1 m^3^/m^2^) | 1.15 (0.69–1.91) | 0.603 | 0.95 (0.55–1.63) | 0.846 | 2.05 (0.75–5.65) | 0.163 |
| 3D volume of abdominal TF (per 1 m^3^/m^2^) | 1.04 (0.97–1.11) | 0.309 | 1.02 (0.94–1.10) | 0.641 | 1.11 (0.96–1.28) | 0.154 |
| 3D volume of abdominal VF (per 1 m^3^/m^2^) | 1.09 (0.92–1.30) | 0.325 | 1.05 (0.87–1.27) | 0.594 | 1.25 (0.82–1.92) | 0.298 |
| 3D volume of abdominal SF (per 1 m^3^/m^2^) | 1.05 (0.95–1.17) | 0.339 | 1.02 (0.91–1.15) | 0.698 | 1.17 (0.96–1.42) | 0.127 |
| Body mass index (per 1 kg/m^2^) | 1.01 (0.98–1.04) | 0.516 | 1.01 (0.98–1.04) | 0.705 | 1.08 (1.01–1.16) | 0.023 |

*Adjusted for age, sex, and variables which had *P* < 0.1 in univariate analysis (deceased donor, pre-transplant dialysis duration, ABO incompatibility, mismatch of human leukocyte antigen, and the induction agents).

Abbreviations: HR, hazard ratio; CI, confidence interval; TF, total fat; VF, visceral fat; SF, subcutaneous fat.

# Supplementary Figures

**Figure S1**. Receiver operating characteristic curves in predicting the 3-year risk of posttransplant diabetes mellitus according to the fat parameters. VF, visceral fat; SF, subcutaneous fat; BMI, body mass index.

**Figure S2**. Cumulative predictive probabilities for the risk of posttransplant diabetes mellitus in 2D waist (A), 3D waist (B), 3D abdominal fat volumes (C), and body mass index (D). AUROC, area under the receiver operating characteristics curve; VF, visceral fat; SF, subcutaneous fat; TF, total fat; BMI, body mass index.


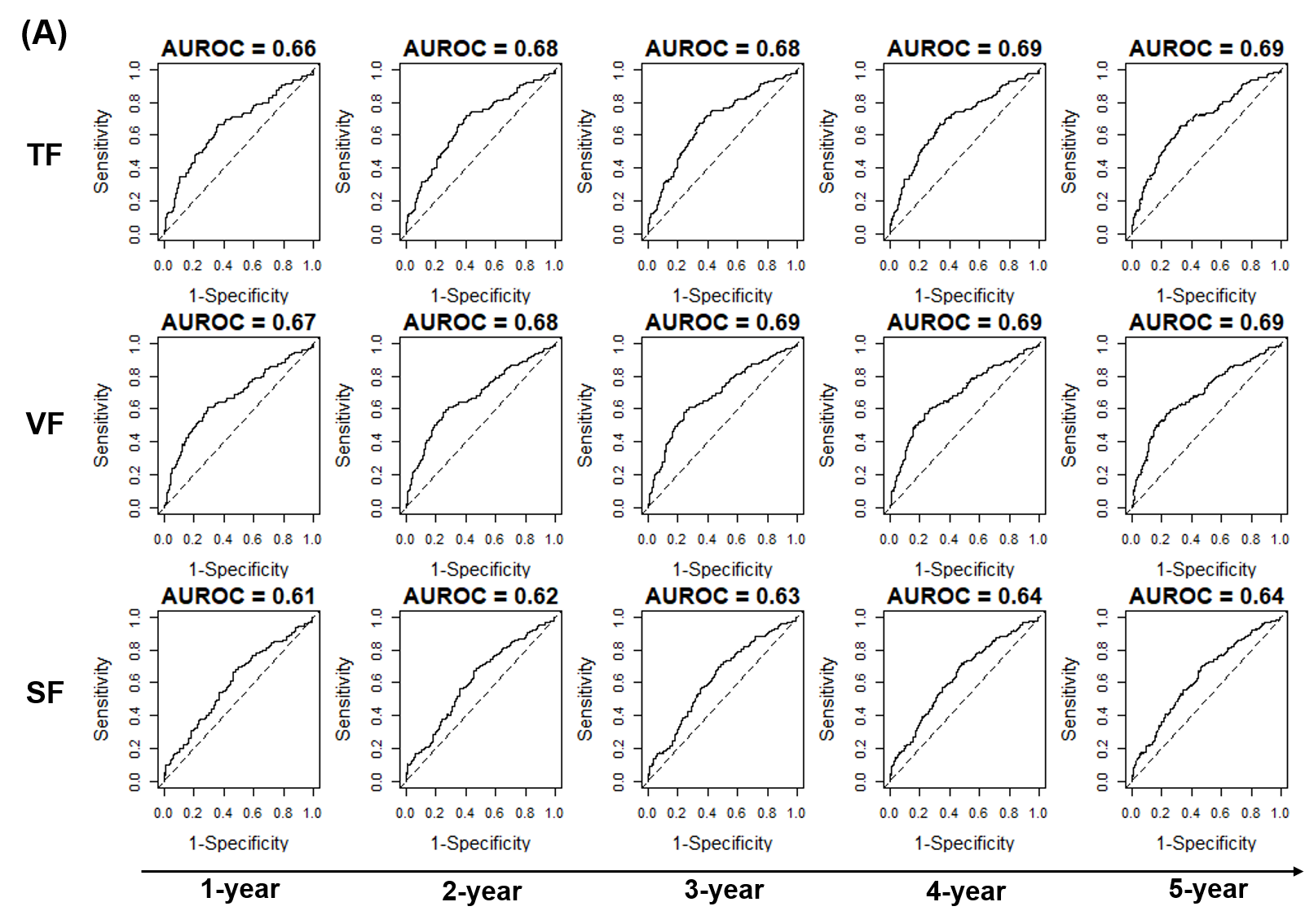


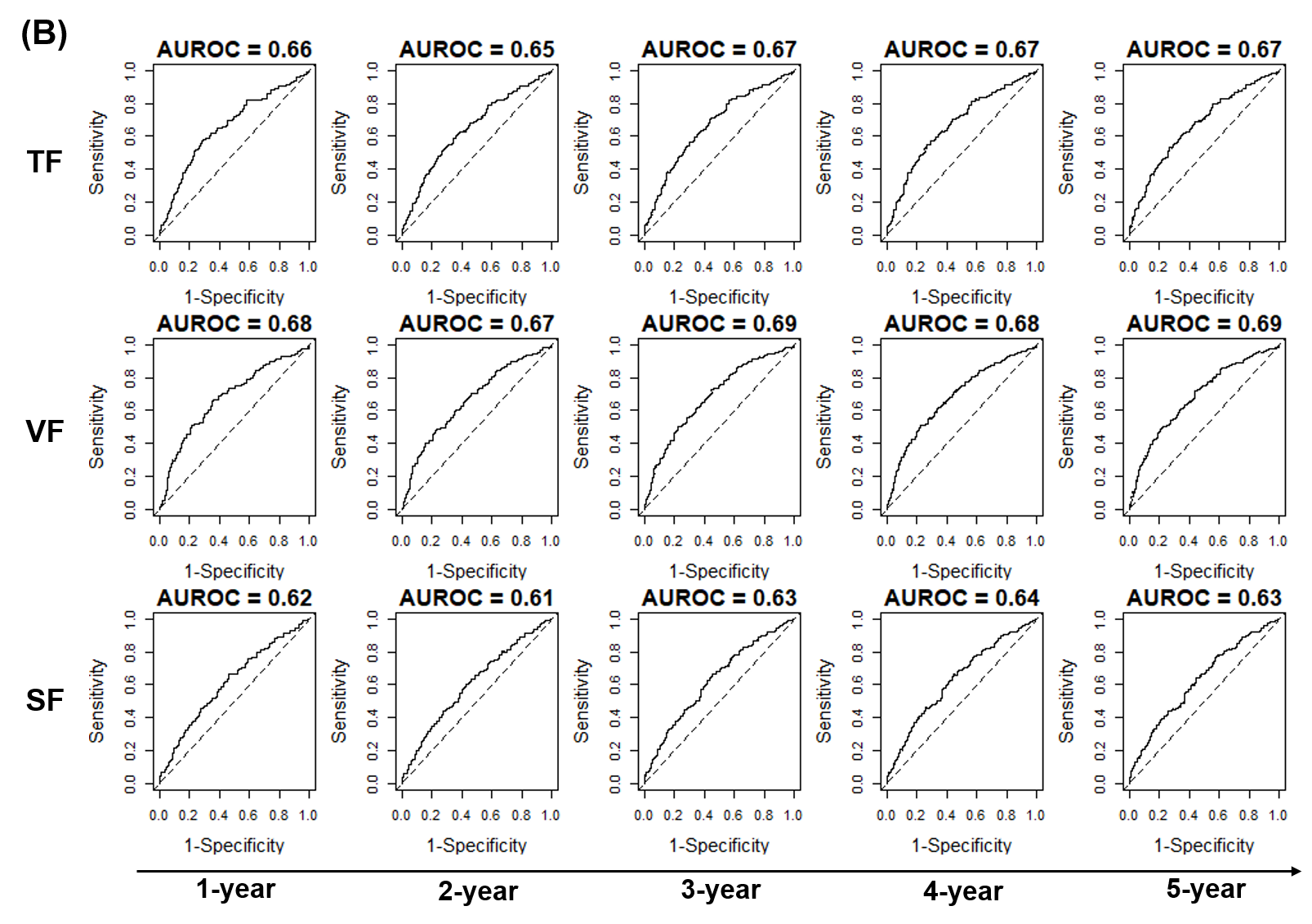


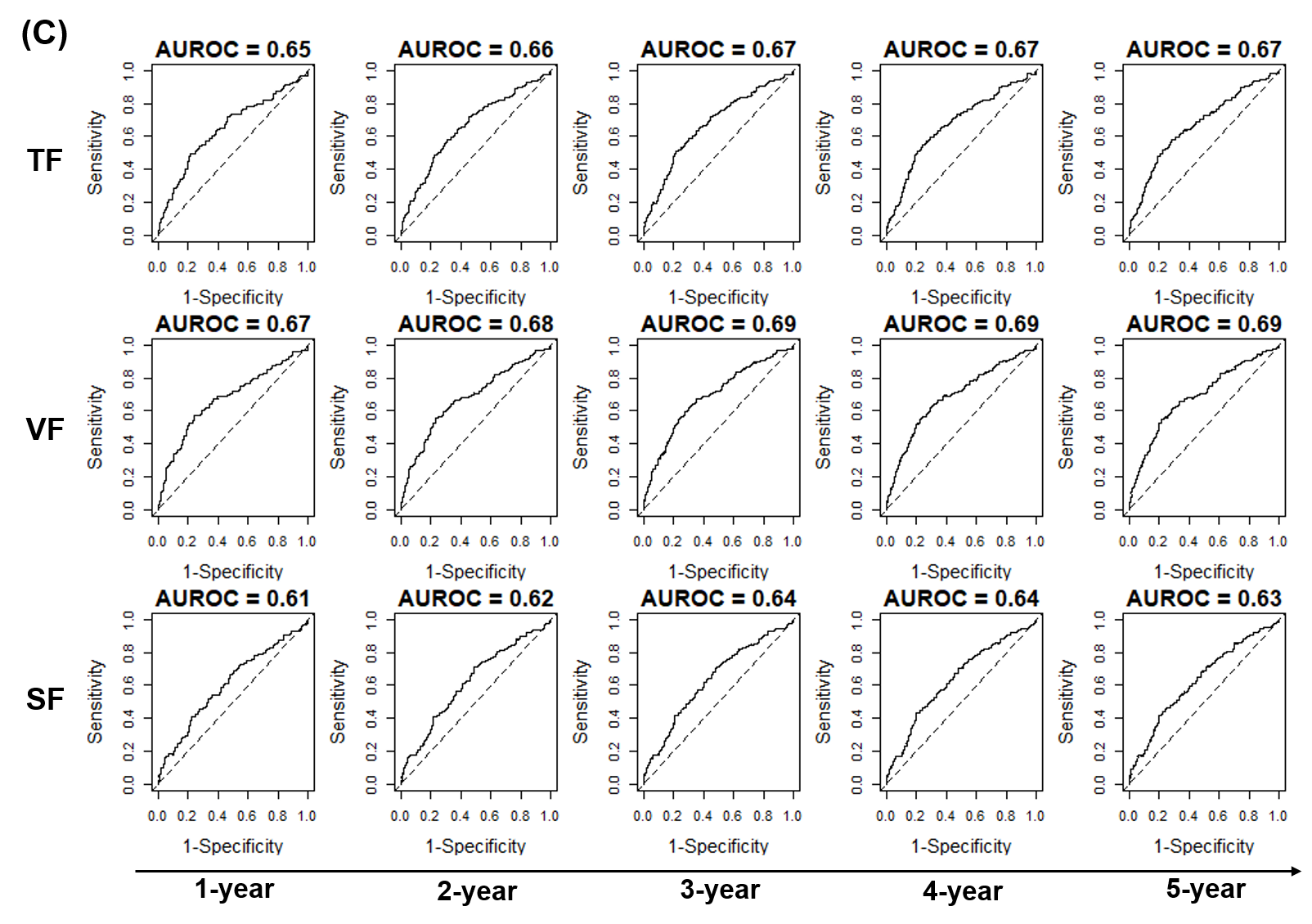


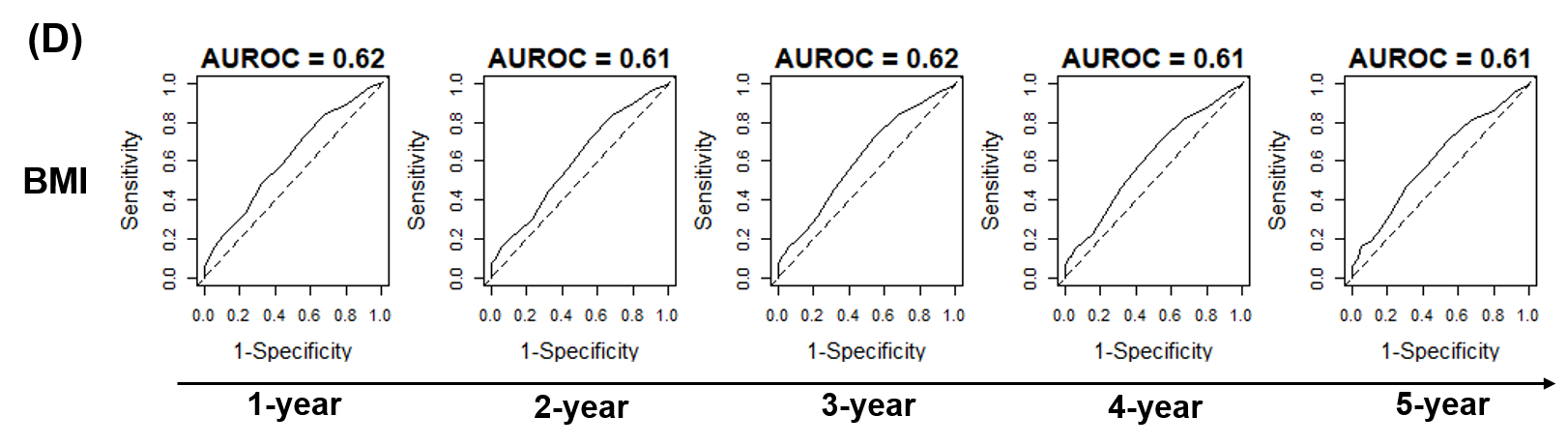

Supplement: Supplementary file 1 [file Data_Sheet_1.docx]
